# Sexual-orientation disparities in whole person health: age- and gender-stratified analysis of patients in a safety-net system

**DOI:** 10.1186/s12939-025-02672-3

**Published:** 2025-11-13

**Authors:** Dhruv Khurana, Maryah Garner, Brittany Bass, Bijan Sasininia, Geoffrey Leung, Anthony Firek

**Affiliations:** 1 Department of Psychiatry and Biobehavioral Sciences, UCLA David Geffen School of Medicine, Los Angeles, CA USA; 2 Department of Health Policy & Management, UCLA Fielding School of Public Health, Los Angeles, CA USA; 3 Comparative Effectiveness and Clinical Outcomes Research Center, Riverside University Health System-Medical Center, Riverside County, Moreno Valley, CA USA; 4 Department of Public Health, Riverside County, Moreno Valley, CA USA; 5 Department of Family Medicine, Riverside University Health System, Moreno Valley, CA USA

**Keywords:** Sexual orientation, LGBTQ, Lesbian, gay, bisexual, Sexual and gender minorities, Health disparities, Patient-reported outcomes, Electronic health records, SOGI, Whole person health score, Safety-net health system, C21, I14

## Abstract

**Introduction:**

Despite advancements in LGB+ rights, LGB+ individuals continue to face significant healthcare disparities, particularly in areas such as homelessness, mental health, substance use, health service access, and victimization. Addressing these disparities is essential to improving the quality of life and health outcomes for this population. This study examines the comprehensive health needs of LGB+ individuals using a Whole Person Health (WPH) approach, which includes Social Determinants of Health (SDOH) as well as other critical aspects of well-being, through EHR-based Sexual Orientation and Gender Identity (SOGI) data within a safety-net health system.

**Methods:**

This cross-sectional study utilizes the EHR-embedded Whole Person Health Score (WPHS) and SOGI tools to assess the SDOH needs of 34,423 heterosexual, 1,213 LGB+, and 384 Other patients. Transgender and gender-fluid patients were excluded for a separate study. The WPHS incorporates self-reported factors across six domains, providing letter grades converted to numerical values and color-coded intervention levels. Data from August 2020 to July 2023 were analyzed.

**Results:**

Relative to heterosexual peers, LGB+ adults showed consistently higher emotional-health burdens - depression (18–25: +24.1 pp, 95% CI 18.7–29.6; 26–45: +17.0 pp, 13.2–20.9; 46+: +7.2 pp, 1.0–13.3) and anxiety (18–25: +22.9 pp, 18.0–27.8; 26–45: +16.6 pp, 13.0–20.3; 46+: +10.1 pp, 4.1–16.2), with similar elevations by gender (e.g. cis female LGB+ depression +18.0 pp, 14.3–21.6). Social adversity was higher across ages, inadequate finances (18–25: +21.5 pp, 16.0–27.1; 26–45: +13.3 pp, 9.6–17.0), food access (+16.0, +10.8, +6.8 pp for 18–25, 26–45, 46+, respectively), and transportation (+11.6 and +10.5 pp for 18–25 and 26–45). Utilization rose for office visits (26–45: +7.4 pp, 3.5–11.4) and prescription use (18–25: +13.5 pp, 7.8–19.2; 26–45: +12.5 pp, 8.7–16.3; 46+: +5.7 pp, 1.9–9.6). Notably, LGB+ adults were more educated (lower “lack of higher education”: 26–45 −11.5 pp, −15.4 to −7.5; 46 + −24.3 pp, −30.4 to −18.1; male LGB+ −22.1 pp, −26.4 to −17.7). Physical-condition differences were mixed (e.g. male LGB+ fewer chronic conditions −10.1 pp, −14.4 to −5.7; fewer functional limitations −13.1 pp, −16.7 to −9.4).

**Conclusion:**

The WPHS tool highlighted substantial disparities in mental health, substance use, and economic challenges among LGB+ individuals, emphasizing the importance of tailored interventions. Our findings advocate for the routine collection of SOGI data and integrating social and non-medical needs in patient care to effectively address these disparities.

## Introduction

There is now an understanding that a person’s overall health, quality of life, and well-being are largely determined by factors beyond genetics, medical determinants, and health services. These factors, collectively known as the Social Determinants of Health (SDOH), play a crucial role in the overall health of patients and communities [1, 2]. This realization has expanded the scope of healthcare delivery to focus on modifiable non-medical factors, such as conditions in which people are born, grow, live, work, and age, and the broader set of forces that shape everyday life [3].

Furthermore, this recognition of SDOH has given rise to a relatively new approach known as Whole Person Health, which focuses on restoring health, promoting resilience, and preventing diseases throughout a person’s lifespan [4].

In Whole Person Health, healthcare providers and systems consider SDOH and emphasize personal behaviors, mental and emotional resilience, community connections, and holistic lifestyle choices that impact health over a lifetime [5]. This approach encourages preventive and rehabilitative interventions that support long-term health maintenance, shifting from merely treating symptoms or managing diseases to addressing the underlying factors contributing to overall health, fostering resilience, and improving quality of life [6]. By expanding beyond SDOH, Whole Person Health promotes personalized and community-centered care, empowering individuals and communities to take active roles in their health through preventive care, mental health support, and lifestyle adjustments [7, 8]. This dynamic and inclusive model of care acknowledges the interconnections between an individual’s internal capacities and external conditions, aiming for sustainable, lifelong health improvements [9].

For marginalized communities, significant gaps in Whole Person Health measures contribute to disparities in health and well-being. Lesbian, Gay, Bisexual, and Non-Heterosexual (LGB+) and transgender individuals, in particular, face unique health challenges and SDOH needs [10]. Traditional models for health care delivery have historically overlooked the unique challenges within these communities. Recognizing this gap, health equity researchers have begun collecting data on Sexual Orientation and Gender Identity (SOGI) to discover information that can help address healthcare disparities in this minority population [11–14].

Conceptual frameworks such as the socio-ecological model, which recognizes the interplay between individual, interpersonal, community, and societal factors [15–17], and the minority stress theory, which describes how societal stressors affect minority health, are particularly relevant here [18, 19]. These frameworks guide the present study in examining how structural and social factors interact with internal resilience and lifestyle behaviors, which is central to the Whole Person Health approach [15–19]. This lens is especially useful in understanding the health challenges faced by LGB+ individuals, who are disproportionately impacted by SDOH-related barriers and may also experience minority stress that further complicates health outcomes. There is a growing literature using representative and population-based data assessing the social determinants of health outcomes among LGB+ populations compared to heterosexual populations. Schuler, Prince, and Collins [20] characterize disparities between LGB adults and heterosexual adults across multiple social health determinants using data from the 2015–2018 National Survey on Drug Use and Health (NSDUH). The authors found that all LGB subgroups exhibited disparities in social determinants of health and were more likely to live alone, to have never been married, and to report low religious service attendance compared to heterosexual adults. Downing and Rosenthal [21] assessed the prevalence of social determinants of health among sexual minority men and women in 2017 using data from the Behavioral Risk Factor Surveillance System (BRFSS). Comparing sexual minorities to heterosexuals, the authors determined that food, housing, and financial insecurity among sexual minorities were greater than those of heterosexuals.

Gonzales and Lavey [10] use community-informed, representative data from 2018 to 2019 to study the SDOH of LGBT adults in Nashville and Davidson County, Tennessee. Compared to non-LGBT adults, LGBT adults reported deficiencies in social support and connectedness. These are critical factors that place LGBT individuals at higher risk for mental health disorders, worsening medical conditions, and increased mortality.

Based on the unmet need to determine the importance of SDOH in this community, the current study employs the Whole Person Health model to understand the collective factors beyond only the SDOH needs of LGB+ individuals using a designated holistic assessment tool, the Whole PERSON Health Score (WPHS), specifically, physical and emotional health, resource utilization, ownership of one’s health, and nutrition and lifestyle factors. To our knowledge, this is the first paper to do so. Accordingly, we compare WPHS profiles of LGB+ and heterosexual patients in a large safety-net system and describe heterogeneity across key subgroups.

Importantly, there is no broad federal requirement to collect sexual orientation and gender identity (SOGI) data. EHR certification emphasizes capability rather than capture, and recent federal policy shifts have introduced enforcement discretion and slowed the release of some equity-relevant data [22]. These gaps highlight the value of system-level, person-reported data, such as WPHS, in characterizing needs that may be undercounted in federal surveillance.

In the following sections, we will explore the methods, data, and results of our study, discuss key findings in the context of existing research, and provide recommendations for integrating Whole Person Health principles to bridge gaps in healthcare for LGB+ populations.

## Methods

This observational, cross-sectional study identified LGB+ individuals within the Riverside University Health System (RUHS) patient population by analyzing data from Epic, RUHSs electronic health record (EHR) system. The study leveraged demographic and holistic health information to assess factors captured by the Whole PERSON Health Score (WPHS) tool, which encompasses Social Determinants of Health (SDOH) and broader dimensions such as mental, emotional, and social well-being. The patient’s sexual orientation was self-reported using Epic’s Sexual Orientation and Gender Identity (SOGI) tool.

The RUHS Human Research Subjects Protection Committee classified the study as exempt, as data were de-identified and collected through routine care processes aimed at identifying and addressing the comprehensive needs of each patient.

### Whole PERSON health score (WPHS)

#### Development & design

In 2022, the authors of this study introduced the Whole *PERSON* Health Score (WPHS), a patient-centered tool designed to measure and address modifiable, nonmedical determinants of overall well-being [23]. The tool design process began with a literature review to identify modifiable factors influencing well-being and longevity. This list was then refined through a multidisciplinary collaborative process that involved input from patients and healthcare professionals, ultimately resulting in 28 elements/questions across the six domains (PERSON). Following local and external validation, the tool was subsequently integrated into RUHSs EHR system (Epic).

The six domains—Physical Health, Emotional Health, Resource Utilization, Socioeconomics, Ownership, and Nutrition & Lifestyle—are collectively referred to as PERSON and designed to capture a holistic view of patient health. The physical health domain evaluates the patient’s current physical condition, including any existing medical conditions. The emotional health domain assesses mental, emotional, and spiritual well-being, whereas the resource utilization domain measures the patient’s engagement with the healthcare system. The socioeconomics domain evaluates factors such as housing, food access, transportation, employment, and financial stability. The ownership domain gauges the patient’s perception of their health, sense of control, confidence, and capacity for positive change. Lastly, the nutrition & lifestyle domain assesses lifestyle habits, including diet, exercise, and sleep quality. Appendix Table A1 provides a comprehensive list of dimensions within each domain. The WPHS is intentionally comprehensive, encompassing more questions and broader domains than most SDOH assessment tools. Unlike standard SDOH tools, which primarily comprise the socioeconomics domain, WPHS is a holistic measure adaptable across sectors and disciplines.

**Fig. 1:**
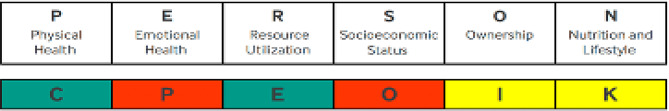
A sample of the PERSON score generated from a completed whole PERSON health score assessment displayed in the patient’s MyChart in epic. This hypothetical score shows that the individual is in critical need of intervention in the emotional health and socioeconomic domains based on red scoring, moderate need for intervention in the ownership and nutrition & lifestyle domains with “yellow” zone, and little/low need for intervention in the physical health and resource utilization domains based on “green” zone score [23]

Each question is scored on a scale of 0 to 6 or 8 (depending on the question), with the scores summed for each domain and converted into an overall letter grade, ranging from A (best) to Z (worst). This letter-grade system simplifies interpretation, promoting patient engagement and understanding of their results. For the care team, the letter grades are further categorized into three color-coded (traffic light system) ratings: Green, Yellow, and Red signaling the need for intervention, where the Red rating indicates the most urgent need for intervention, green indicating no need at the moment. A sample score is illustrated in Figure 1 below (screenshot from Epic).

It is essential to note that these ratings are generated based on patient-reported information, ensuring that the needs identified are centered around the patient’s perspective rather than the clinicians’.

#### Validation

The WPHS underwent a comprehensive validation process to ensure its reliability and applicability. Content validity was established through expert input and integrating elements from validated instruments like PHQ-9 (Patient Health Questionnaire 9-item), GAD-7 (Generalized Anxiety Disorder 7-item), SHA (Staying Healthy Assessment), SBIRT (Screening, Brief Intervention, and Referral to Treatment), and AUDIT (Alcohol Use Disorders Identification Test). Convergent validity showed strong correlations with PHQ-9/GAD-7 and change scores (*r* ≈ 0.7) in emotional health, resource utilization, and ownership; structural validity via factor analysis (loadings > 0.4); criterion validity via links to BMI, blood pressure, comorbidities, and care outcomes (labs, appointment adherence); and discriminant validity via weak ties to unrelated metrics. Ecological validity was supported by alignment with operational health system metrics, ensuring its real-world utility.

Please refer to Khurana et al. [23] for a detailed description of the WPHS tool’s design, implementation, validation, and utility.

#### Scope of measurement

The WPHS is designed to identify person-level, modifiable needs (e.g., housing instability, food access, mental health symptoms, self-management/ownership). It does not directly measure upstream structural determinants of health (e.g., historical housing policy, environmental burden, policing intensity, payer network design, prior-authorization policies) or organizational/clinical norms (e.g., language-concordant care, interpreter availability/quality, appointment length/productivity norms, perceived discrimination/medical mistrust).

### Study sample

We used WPHS assessment data collected from August 2020 to July 2024 for the analysis. During this period, 93,000 assessments were started, with 15,545 classified as incomplete. Our analysis included only completed patient WPHS assessments, totaling 77,455 assessments (83.3%), which were completed by 48,641 unique patients. Only the final completed assessments were used, as a score can only be generated from completed WPHS assessments. Of the 48,641 unique patients, we restricted our analysis to those 18 years or older and to patients who identified as either cis male or cis female. Our final sample included 48,549 patients, of whom 12,557 (25.9%) did not report any sexual orientation and were thus excluded from the analysis. Of the remaining 35,992 patients, 34,397 (95.6%) identified as Heterosexual (or Straight), 1,212 (3.37%) identified as LGB+ (Lesbian, Gay, Lesbian or Gay, Bisexual, Pansexual, Asexual, Something else), and 383 (1.06%) were categorized as “Other,” including those who answered, “Choose not to disclose” and “Do not Know” to the sexual orientation question during intake. The SOGI data was collected separately in Epic and merged with the individual WPHS assessments. We included the “Other” group in the analysis, as their data helped us understand variations in the needs of patients who did not fall into the traditional heterosexual or LGB+ categories. We did not include patients who self-identified as transgender a priori, as their unique needs would be measured in a separate study. We also recategorized the race and ethnicity categories to align with the US Census Bureau-approved categories [24]. Due to the sparse cell counts after cross-stratifying sexual orientation and race/ethnicity, we did not conduct race/ethnicity-stratified estimates; intersectional analyses are planned once sample sizes permit.

### Statistical analysis

Using the patients’ individual WPHS scores, we identified the patients’ needs as a binary variable, classifying Red zone scores as “critical” and Yellow or Green scores as “not critical” for each domain. Using this binary outcome, we employed logistic regression analysis to investigate whether individuals within the LGB+ community exhibited a higher probability of experiencing critical needs (Red zone) within five of the six WPHS domains compared to their heterosexual counterparts. Notably, there was a very low incidence of critical scores in physical health (1.4% − 503 out of 35,992, with only 20 identifying as LGB+), so we excluded physical health from the analysis.

To examine how sexual orientation affects outcomes differently based on patient gender and age groups, we created three distinct age brackets: 18–25 (young adults), 26–45 (adults), and 46 and above (older adults). For gender, only patients identifying as cis male or cis female were included in the analysis.

We also utilized the comprehensive and granular nature of the WPHS assessment to understand better the factors contributing to differences in critical needs within each domain. We created a binary variable for each of the 28 dimensions of the WPHS that indicated an area of concern (e.g., “felt depressed in the past month”). Table 1 presents a complete list of the dimensions within each domain. Logistic regression models were employed to determine statistical differences in the probability of having an unfavorable outcome for each dimension within every domain, comparing LGB+ patients with their heterosexual counterparts. It is important to note that an adverse outcome in a single dimension does not necessarily lead to an overall critical need for the domain; instead, a critical need requires a patient to meet the threshold for multiple unfavorable outcomes within a domain.

**Table 1:** Demographic breakdown of the study sample by sexual orientation

Characteristics	Overall		Heterosexual		LGB+		Other	
	N	%	N	%	N	%	N	%
	35,992	100%	34,397	100%	1,212	100%	383	100%
**Age**								
18–25	3,968	11.00%	3,575	10.40%	314	25.90%	79	20.60%
26–45	14,159	39.30%	13,353	38.80%	642	53.00%	164	42.80%
46+	17,866	49.60%	17,469	50.80%	256	21.10%	140	36.60%
**Biological Sex**								
Female	23,595	65.60%	22,692	66.00%	685	56.50%	218	56.90%
Male	12,397	34.40%	11,705	34.00%	527	43.50%	165	43.10%
**Race & Ethnicity**								
Hispanic	23,579	65.50%	22,757	66.20%	648	53.50%	174	45.40%
Non-Hispanic Asian	1,996	5.50%	1,899	5.50%	71	5.90%	26	6.80%
Non-Hisp. American Indian/Alaskan Native	90	0.30%	81	0.20%	9	0.70%	0	0.00%
Non-Hispanic Black	2,962	8.20%	2,781	8.10%	135	11.10%	46	12.00%
Non-Hisp. Native Hawaiian/Pacific Islander	92	0.30%	84	0.20%	3	0.20%	5	1.30%
Non-Hispanic White	6,623	18.40%	6,200	18.00%	304	25.10%	119	31.10%
Others	124	0.30%	116	0.30%	6	0.50%	2	0.50%
Ethnicity Unknown	526	1.50%	479	1.40%	36	3.00%	11	2.90%

Additionally, we report the average marginal effects (AMEs) instead of odds ratios (ORs) from logistic regression to improve the clarity and interpretability of results, especially for applied research audiences. While ORs describe changes in the odds of an event, they are often misinterpreted as changes in probabilities or relative risks, which can lead to confusion [25]. Marginal effects provide a direct and intuitive interpretation of how changes in predictors influence the probability of an outcome [26]. Unlike ORs, marginal effects can vary across subgroups, enabling a more nuanced understanding of heterogeneity in treatment effects [27]. They facilitate comparisons between subgroups or populations, which is critical in addressing health disparities and designing equitable policies. Further, Norton, Dowd, Garrido, and Maciejewski [28] advocate for the routine reporting of marginal effects in health services research to improve the accessibility, accuracy, and policy relevance of statistical findings, given that health services researchers are often interested in the magnitude of effects to assess policy relevance, rather than simply the direction of the association.

This paper focuses on the most critical needs, represented by the Red zone scores across the six domains. The decision to prioritize interventions for critical needs reflects a strategic and targeted approach to resource allocation, aiming to address the most pressing issues. The critical needs represent areas that, if left unaddressed, could lead to significant adverse impacts on patient well-being in the respective domains. The data were analyzed using R and STATA MP-17 software. These tools were chosen due to their robust capabilities for data cleaning, regression analysis, and efficient handling of EHR datasets.

## Results

Based on the results presented in Table 1, across age groups, half of all Heterosexuals are ages 46+ (50.8%), while nearly half of all LGB+ (53%) and Others (42.8%) are ages 26 to 45 years. Over half of all WPHS respondents identified as cis Female across all sexual orientation groups (LGB+: 56.5% to Heterosexual: 66%).

Table 2 shows that LGB+ individuals fare worse than heterosexuals across all six WPHS domains, with a larger percentage of LGB+ individuals in the Red Zone compared to heterosexuals. The differences are particularly notable in the Emotional Health, and Nutrition & Lifestyle domains. In the Emotional Health domain, 20.1% of LGB+ individuals required critical intervention (Red Zone) compared to only 9.8% of heterosexuals. Similarly, 19.7% of LGB+ patients were in the Red Zone for Nutrition & Lifestyle, compared to 12.7% of heterosexual patients. These disparities are evident in the raw distributions presented in Table 2.

**Table 2:** Distribution of the PERSON score results for each zone: red, yellow, and Green, by sexual orientation

Characteristics	Overall		Heterosexual		LGB+		Other	
	N	%	N	%	N	%	N	%
**Physical Health**								
Red	503	1.40%	480	1.40%	20	1.60%	3	0.80%
Yellow	17,737	49.30%	16,970	49.30%	569	46.90%	198	51.70%
Green	17,752	49.30%	16,947	49.30%	623	51.40%	182	47.50%
**Emotional Health**								
Red	3,670	10.20%	3,372	9.80%	243	20.10%	55	14.30%
Yellow	16,139	44.80%	15,368	44.70%	590	48.60%	181	47.10%
Green	16,183	45.00%	15,647	45.50%	379	31.20%	147	38.50%
**Resource Utilization**								
Red	2,211	6.10%	2,084	6.10%	89	7.30%	38	9.90%
Yellow	11,990	33.30%	11,488	33.40%	373	30.80%	129	33.60%
Green	21,791	60.50%	20,825	60.60%	750	61.90%	216	56.50%
**Socioeconomics**								
Red	3,162	8.80%	2,960	8.60%	157	12.90%	45	11.70%
Yellow	21,630	60.10%	20,663	60.10%	733	60.50%	234	61.20%
Green	11,200	31.10%	10,774	31.30%	322	26.50%	104	27.10%
**Ownership**								
Red	2,404	6.70%	2,273	6.60%	96	7.90%	35	9.10%
Yellow	25,320	70.40%	24,211	70.40%	851	70.20%	258	67.40%
Green	8,268	23.00%	7,913	23.00%	265	21.80%	90	23.40%
**Nutrition & Lifestyle**								
Red	4,529	12.60%	4,224	12.30%	239	19.70%	66	17.20%
Yellow	26,283	73.00%	25,169	73.20%	850	70.20%	264	69.00%
Green	5,180	14.40%	5,004	14.60%	123	10.10%	53	13.80%

### Age-based disparities (Table 3, Fig. 2, Appendix table A2)

#### Young LGB+ v. Young heterosexual patients (18–25 years)

Based on the results presented in Table 3, among younger patients (18–25 years), LGB+ individuals showed significantly poorer outcomes in several domains compared to their heterosexual peers. They were more likely to experience adverse Emotional Health (17.4% points, or pp, 95% CI [12.3, 22.5]), Socioeconomic (7.1 pp, 95% CI [3.7,10.5]), and Nutrition & Lifestyle (9.4 pp, 95% CI [5.3,13.5]) outcomes. However, no statistically significant differences were noted in the Physical Health domain.

**Fig. 2:**
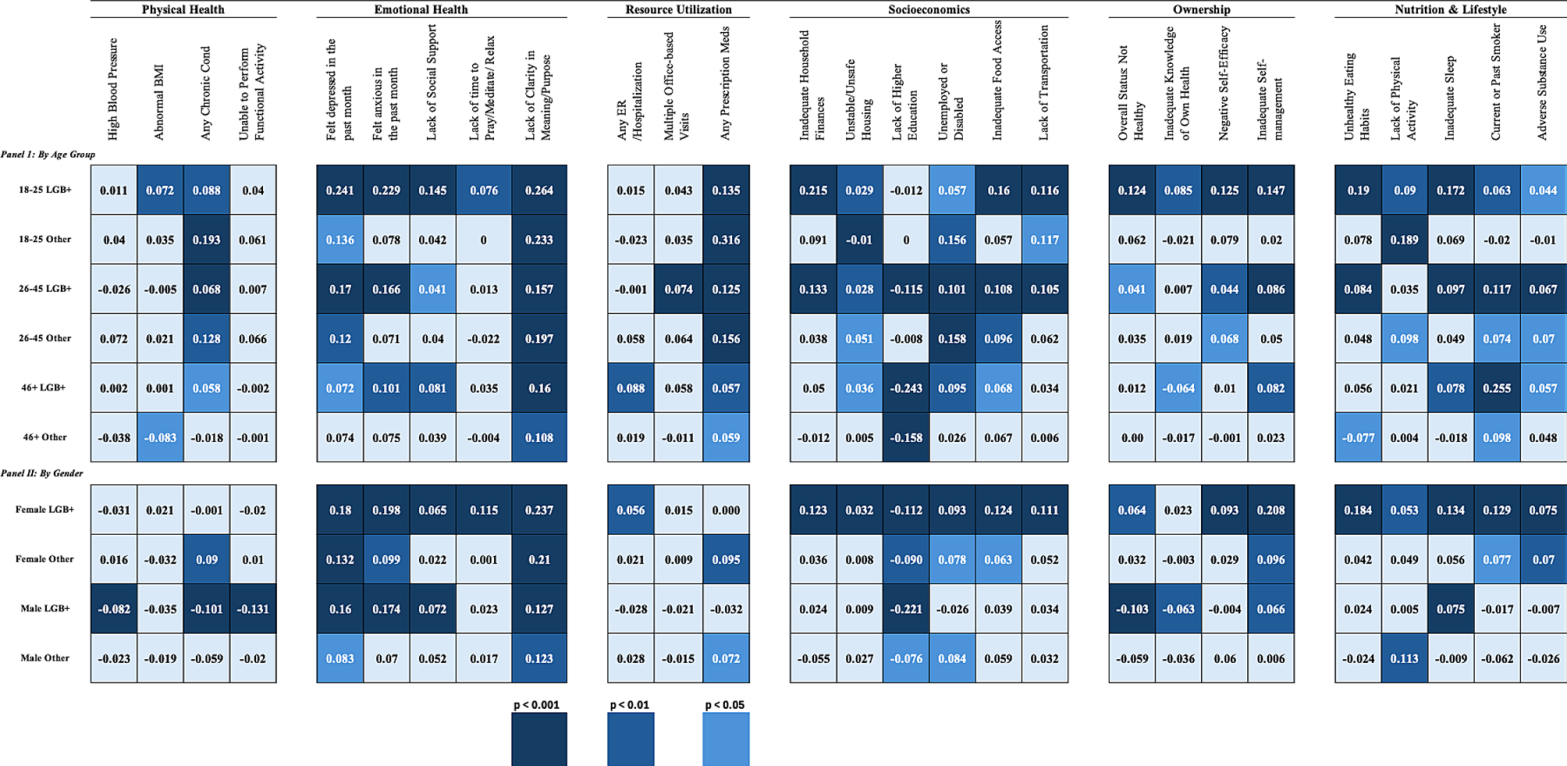
Heatmap showing the average marginal effect (difference in percentage points) between LGB+ and their heterosexual counterparts by age groups and gender across 28 dimensions. Darker fills represent a higher level of statistical significance. Interpretation: young LGB+ individuals reported higher rates of depression (24.1% points, or pp) and anxiety (22.9 pp) compared to young heterosexual patients

**Table 3:** Average marginal effects of the disparities in the probability of scoring in the “red” zone by Domain among LGB+ and others by age group and gender. Notes: the reference group is heterosexual individuals of the same age or gender. Each column in each panel is a separate regression. Average marginal effects are presented. Standard errors are presented in parentheses, and 95% confidence intervals are presented in brackets

	**Physical Health**	**Emotional Health**	**Resource Utilization**	**Socioeconomics**	**Ownership**	**Nutrition & Lifestyle**
**Panel 1: By Age Group**						
18–25 LGB+	0.001	0.174***	0.016	0.071***	0.026+	0.094***
	−0.003	−0.026	−0.012	−0.018	−0.015	−0.021
	[−0.005,0.008]	[0.123,0.225]	[−0.007,0.039]	[0.037,0.105]	[−0.004,0.056]	[0.053,0.135]
18–25 Other	−0.001**	0.046	0.013	0.042	0.038	0.09*
	−0.0007	−0.041	−0.022	−0.03	−0.032	−0.041
	[−0.003, −0.0005]	[−0.034,0.125]	[−0.030,0.055]	[−0.017,0.101]	[−0.025,0.101]	[0.007,0.166]
26–45 LGB+	−0.0022	0.078***	0.018+	0.066***	0.022*	0.08***
	−0.0027	−0.015	−0.01	−0.014	−0.011	−0.016
	[−0.007, 0.003]	[0.048,0.108]	[−0.001,0.037]	[0.039,0.092]	[0.000,0.043]	[0.049,0.112]
26–45 Other	−0.0008	0.037	0.084**	0.067**	0.055*	0.075*
	−0.006	−0.027	−0.026	−0.027	−0.025	−0.031
	[−0.013,0.011]	[−0.016,0.091]	[0.033,0.135]	[0.015,0.120]	[0.006,0.104]	[0.014,0.136]
46+ LGB+	0.04**	0.060**	0.060**	0.041+	0.001	0.098***
	−0.015	−0.023	−0.022	−0.023	−0.017	−0.027
	[0.011,0.070]	[0.015,0.104]	[0.018,0.103]	[−0.003,0.085]	[−0.032,0.033]	[0.046,0.150]
46+ Other	−0.007	0.050+	0.02	0.01	−0.009	0.021
	−0.01	−0.03	−0.025	−0.028	−0.021	−0.031
	[−0.027,0.012]	[−0.008,0.108]	[−0.030,0.070]	[−0.044,0.064]	[−0.050,0.032]	[−0.040,0.081]
*N*	35,992	35,992	35,992	35,992	35,992	35,992
**Panel II: By Gender**						
Female LGB+	0.002	0.125***	0.022*	0.065***	0.032**	0.110***
	−0.005	−0.016	−0.01	−0.013	−0.011	−0.016
	[−0.007,0.012]	[0.093,0.156]	[0.002,0.042]	[0.039,0.090]	[0.010,0.054]	[0.079,0.141]
Female Other	−0.004	0.057*	0.042*	0.032	0.03	0.074**
	−0.007	−0.024	−0.02	−0.02	−0.02	−0.026
	[−0.017,0.008]	[0.009,0.105]	[0.003,0.082]	[−0.007,0.072]	[−0.009,0.068]	[0.024,0.125]
Male LGB+	0.003	0.071***	−0.003	0.002	−0.014	0.014
	−0.006	−0.017	−0.011	−0.015	−0.011	−0.017
	[−0.009,0.014]	[0.038,0.104]	[−0.026,0.019]	[−0.027,0.031]	[−0.034,0.007]	[−0.020,0.047]
Male Other	−0.008	0.028	0.029	0.017	0.017	0.003
	−0.006	−0.027	−0.024	−0.028	−0.023	−0.029
	[−0.021,0.004]	[−0.024,0.081]	[−0.017,0.076]	[−0.038,0.071]	[−0.027,0.061]	[−0.054,0.061]
*N*	35,992	35,992	35,992	35,992	35,992	35,992

*** indicates *p* < 0.001, ** *p* < 0.01, * *p* < 0.05, + *p* < 0.1

Based on the heatmap presented in Fig. 2, specifically, young LGB+ individuals reported higher rates of depression (24.1 pp, 95% CI [18.7, 29.6]), anxiety (22.9 pp, 95% CI [18, 27.8]), and chronic conditions (8.8 pp, 95% CI [3.4, 14.2]). They also faced greater financial instability, in terms of household finances (21.5 pp, 95% CI [15.9, 27.1]), unemployment or disability (5.7 pp, 95% CI [0.3, 11]), food insecurity (16 pp, 95% CI [10.6, 21.4]), and adverse lifestyle factors, including unhealthy eating habits (19.0 pp, 95% CI [13.3, 24.6]), insufficient physical activity (9 pp, 95% CI [3.4, 14.6]), smoking (6.3 pp), and substance use (4.4 pp, 95% CI).

#### Middle-aged LGB+ v. middle-aged heterosexual patients (26–45 years)

They were more likely to report urgent need for intervention in Emotional Health (7.8 pp, 95% CI [4.8,10.8]), Socioeconomics (6.6 pp, 95% CI [3.9,9.2]), Nutrition & Lifestyle (8 pp, 95% CI [4.9, 11.2]).

Specifically, based on the heatmap presented in Fig. 2, middle-aged LGB+ individuals (26–45 years) exhibited similar patterns, with significantly higher rates of depression (17.0 pp, 95% CI [13.2, 20.9]), anxiety (16.6 pp, 95% CI [13, 20.3]), and chronic conditions (6.8 pp, 95% CI [2.9, 10.7]). They also had greater financial insecurity (13.3 pp, 95% CI [9.6, 17]), unemployment or disability (10.1 pp, 95% CI [6.4, 13.9]), and unhealthy behaviors such as smoking (11.7 pp, 95% CI [7.9, 15.5]), substance use (6.7 pp, 95% CI 3.5, 9.8]), and poor nutrition (8.4 pp, 95% CI [4.5, 12.4]). Additionally, middle-aged LGB+ individuals were more likely to report negative self-efficacy (4.4 pp, 95% CI [1.1, 7.7]) and inadequate self-management (8.6 pp, 95% CI [4.6, 12.5]).

#### Older aged LGB+ v. older aged heterosexual patients (46+ years)

Older LGB+ patients (46+ years) also showed higher rates of poor Emotional Health (6.0 pp, 95% CI [1.5, 10.4]), Resource Utilization (6.0 pp, 95% CI [1.8, 10.3]), and Nutrition & Lifestyle (9.8 pp, 95% CI [4.6, 15.0]) (refer to Table 3).

Specifically, they were 7.2 pp (95% CI [1.0, 13.3]) more likely to report depression, 10.1 pp (95% CI [4.1, 16.2]) more likely to report anxiety, and 8.1 pp (95% CI [2.6, 13.5]) more likely to report a lack of social support. Additionally, they had 5.8 pp (95% CI [0.2, 11.3]) higher rates of chronic conditions and were 25.5 pp (95% CI [19.4, 31.6]) more likely to report being a current or past smoker and 5.7 pp (95% CI [0.9, 10.4]) more likely to report adverse substance use. In terms of Socioeconomics, older LGB+ individuals were 5.0 pp (95% CI [−0.7, 10.8]) more likely to face financial insecurity, 9.5 pp (95% CI [0.9, 12.6]) more likely to experience unemployment or disability, and 6.8 pp (95% CI [−1.9, 8.6]) more likely to experience food insecurity. However, they were 24.3 pp (95% CI [−30.4, −18.1]) less likely to lack higher education compared to their heterosexual peers (refer to Fig. 2).

### Gender-based disparities (Table 3, Fig. 2, Appendix table A2)

#### Cis female LGB+ v. Cis female heterosexual patients

Cis Female LGB+ patients consistently showed higher rates of poor outcomes across most domains. They were 12.5 pp (95% CI [9.3,15.6]) more likely than heterosexual females to report poor Emotional Health, 6.5 pp (95% CI [3.9,9.0]) more likely to face Socioeconomic challenges, and 11 pp (95% CI [7.9,14.1]) more likely to report poor Nutrition & Lifestyle.

Specifically, in terms of mental health, cis Female LGB+ patients were 18.0 pp (95% CI [14.3, 21.6]) more likely to experience depression and 19.8 pp (95% CI [16.5, 23.2]) more likely to feel anxious. They also reported higher rates of social support deficits (6.5 pp, 95% CI [3.1, 10.0]), lack of time for self-care (11.5 pp, 95% CI [7.7, 15.3]), and lack of clarity in purpose (23.7 pp, 95% CI [20.0, 27.5]). They were 5.6 pp (95% CI [1.8, 9.3]) more likely to visit doctors frequently.

Social determinants of health show that cis Female LGB+ patients were 12.3 pp (95% CI [8.8, 15.9]) more likely to face financial insecurity, 9.3 pp (95% CI [5.7, 13.0]) more likely to be unemployed, and 12.4 pp (95% CI [8.8, 16.0]) more likely to experience food insecurity. However, they were 11.2 pp (95% CI [−15.0, −7.4]) less likely to lack higher education.

Regarding self-perception, they were 6.4 pp (95% CI [2.6, 10.2]) more likely to report poor health, 9.3 pp (95% CI [5.9, 12.7]) more likely to report negative self-efficacy, and 20.8 pp (95% CI [17.0, 24.6]) more likely to struggle with self-management. Finally, cis Female LGB+ patients were 18.4 pp (95% CI [14.7, 22.2]) more likely to report unhealthy eating habits, 13.4 pp (95% CI [10.4, 16.4]) more likely to have insufficient sleep, and 7.5 pp (95% CI [4.8, 10.3]) more likely to have substance use issues (Refer to Fig. 2).

#### Cis male LGB+ v. Cis male heterosexual patients

Cis Male LGB+ patients were 7.1 pp (95% CI [3.8,10.4]) more likely than their heterosexual counterparts to report poor Emotional Health. However, no other significant differences were observed across other domains.

Specifically, cis Male LGB+ patients were 8.2 pp (95% CI [−12.4, −3.9]) less likely to report high blood pressure, 10.1 pp (95% CI [−14.4, −5.7]) less likely to have chronic conditions, and 13.1 pp (95% CI [−16.7, −9.4]) less likely to report difficulty performing any functional activity. They were also 16.0 pp (95% CI [11.7, 20.4]) more likely to experience depression and 17.4 pp (95% CI [13.2, 21.6]) more likely to feel anxious. Additionally, they were 7.2 pp (95% CI [3.3, 11.1]) more likely to report a lack of social support and 12.7 pp (95% CI [8.5, 16.9]) more likely to feel a lack of purpose in life. Regarding resource utilization, cis Male LGB+ patients were 3.2 pp (95% CI [−7.4, 0.9]) less likely to have been prescribed medication (i.e., the effect is negative and not statistically significant). Social determinants of health indicated that cis Male LGB+ patients were 22.1 pp (95% CI [−26.4, −17.7]) less likely to lack higher education, 10.3 pp (95% CI [−14.4, −6.2]) less likely to report poor overall health, and 6.3 pp (95% CI [−10.2, −2.5]) less likely to lack knowledge of their health. However, they were 6.6 pp (95% CI [2.3, 10.9]) more likely to report poor self-management. Lastly, in terms of Nutrition & Lifestyle, cis Male LGB+ patients were 7.5 pp (95% CI [3.5, 11.5]) more likely to report inadequate sleep (refer to Fig. 2).

## Discussion

This study employed the WPHS, an EHR-embedded holistic assessment tool/whole person screener, to examine the specific unmet needs of the LGB+ community, a marginalized group whose health challenges are often overlooked by healthcare providers and policymakers. Prior work has sometimes treated LGB+ needs as interchangeable with those of cisgender heterosexual populations, obscuring distinct challenges [29]. The results emphasize the importance of recognizing and addressing the unique health needs of LGB+ populations to ensure equitable healthcare delivery. As widely documented in the literature, the LGB+ community faces significant medical, mental health, and socio-economic challenges [10]. Our study corroborates these findings, revealing that LGB+ individuals experience higher rates of depression, anxiety, substance use, and economic barriers compared to their heterosexual counterparts [20, 30]. Specifically, our study found that LGB+ patients reported significantly higher levels of depression, anxiety, and inadequate social support [31, 32]. Additionally, they exhibited higher medication usage, poor self-management practices, and insufficient sleep.

Heterogeneity by age and gender was substantial. Younger LGB+ individuals (aged 18–34) faced greater challenges in managing nutrition and lifestyle, along with heightened mental health issues. These young individuals are particularly vulnerable to the compounded effects of discrimination [33], identity formation struggles, and socio-economic instability. For instance, LGB+ youth are more likely to experience bullying, which contributes to emotional stress, suicidal ideation, and self-harm [34]. They are also more likely to experience homelessness than their heterosexual peers [35]. This stress often continues into college years, further contributing to depression and social isolation [36]. Studies show that LGB+ youth are also at higher risk for developing eating disorders [37] and experiencing suicidal thoughts when compared to their heterosexual peers [36]. Further, younger LGB+ individuals, specifically females, are also more likely to report higher substance use [38]. The heightened mental health challenges of LGB+ youth highlight the urgent need for targeted interventions that support their emotional and psychological well-being [39].

In contrast, older LGB+ individuals (aged 46 and above) were found to report poorer mental health, including higher levels of depression and anxiety [40–42], along with significant socio-economic challenges such as financial instability, unemployment, and food insecurity [43]). This group also faces unique barriers, with a history of navigating a less inclusive society, which may limit their access to resources such as higher education and healthcare. Our findings suggest that older LGB+ individuals may benefit from interventions tailored to addressing chronic mental health conditions, improving social support, and alleviating socio-economic stressors. Cis Female LGB+ individuals across all age groups also face broader health and socio-economic challenges compared to their cis Male counterparts [44, 45]. They experience higher levels of mental health issues, including depression and anxiety [45, 46], and also report greater difficulties with access to healthcare, financial stability, and nutrition. In addition, these individuals are more likely to face housing instability and food insecurity, factors that exacerbate their overall well-being [47, 48]. For these reasons, targeted interventions focusing on both mental health care and socio-economic support are crucial for improving outcomes for cis Female LGB+ patients.

Socioeconomic gradients were pronounced. LGB+ individuals, particularly youth, are more likely to experience homelessness due to their sexual identity, which contributes to financial instability and exacerbates long-term socio-economic challenges [49]. Disparities in educational attainment and economic stability were especially evident among lesbian/gay females, with younger individuals more likely to lack a college degree. In comparison, older individuals showed less of this gap. Bisexual females were found to face economic and healthcare access challenges across nearly all age groups, with notable disparities in education, household poverty, unemployment, and lack of health insurance [45].

Interestingly, gay men, while exhibiting some disparities, generally showed advantages in education and economic stability when compared to heterosexual peers [20]. These findings further underscore the importance of addressing socio-economic disparities within LGB+ populations, particularly focusing on education and employment opportunities to alleviate poverty and improve access to healthcare. In terms of nutrition and lifestyle, LGB+ individuals consistently reported higher rates of substance use, including smoking, binge drinking, and illicit drug use [45, 50, 51].

Data were collected post-COVID, when sexual minority disparities likely intensified [52–55]. Additionally, by analyzing individual-level, linked SOGI and whole health data from a large Southern California safety-net health system, we can explore more fundamental challenges within the LGB+ community free from many restrictive and discriminatory policies now emerging in other regions. These policies often overlook the SDOH needs of the LGB+ population, making our findings relevant to the broader national context.

Recent national survey data (e.g., a [56]) show that the percentage of U.S. adults identifying as LGBT+ has doubled since 2012, signaling a growing population that demands increased attention and support [56]. This shift marks a potential new era of inclusion, as more LGB+ individuals feel comfortable identifying openly. However, the increasing visibility of the LGB+ population also highlights the continuing disparities they face in accessing healthcare and other systemic resources crucial for improving health outcomes. As more LGB+ individuals emerge from the shadows, healthcare systems must evolve to meet their unique needs.

To address the health disparities identified in this study, policymakers, healthcare providers, and advocacy organizations should collaborate on strategies that align with a Whole Person Health model, integrating both medical and non-medical aspects of care. This approach goes beyond social determinants of health by emphasizing the mental, emotional, and social aspects of well-being and physical health. Enhancing medical education and provider competency is essential, as current gaps in cultural competency training can lead sexual minorities to avoid necessary care due to fear of discrimination [57]. To support a Whole Person Health approach, healthcare systems must incorporate tools to assess and address socioeconomic stressors, mental health needs, resilience factors, and social support systems. By developing interventions that consider these dimensions of health, we can foster an environment that improves the holistic well-being and health outcomes of LGB+ individuals.

While our study contributes valuable insights into the health disparities faced by LGB+ individuals, it is essential to acknowledge its limitations. The study’s cross-sectional design limits our ability to establish causal relationships, and the sample’s safety-net healthcare setting may not fully represent all LGB+ individuals. Response bias may also be a factor, as individuals with more health-related concerns may have been more likely to participate. WPHS effectively screens proximate, modifiable needs at the individual level. However, inequities observed in these domains are shaped by upstream structures, historical redlining, environmental burden, zoning/land use, policing/fines/fees, and benefit design (e.g., network adequacy, prior authorization) and by organizational/clinical norms, language concordance, interpreter availability/quality, appointment norms, and perceived discrimination/medical mistrust. WPHS does not directly measure these structural or cultural channels, and our analyses were not designed to do so. We did not conduct area-level linkages (e.g., ADI/SVI/EJ indices), redlining overlays, or EHR-based culture measures in this study; thus, results should be interpreted as person-level patterns without direct measurement of neighborhood context or rules and norms.

To that end, for operational deployments, we recommend pairing WPHS (or a similar assessment tool) with place-based context via routine geocoding and attachment of ADI (and jurisdictional EJ indices or historical redlining layers where available) and culture indicators available in the EHR (language concordance; interpreter use). Reporting WPHS outcomes stratified by these contextual indicators can inform place-responsive and culture-responsive quality improvement (e.g., interpreter services, network adequacy escalation, community partnerships in high-deprivation areas). Despite these limitations, our study provides a crucial starting point for understanding the unique needs of LGB+ individuals and offers a foundation for future research addressing health inequities.

Notably, there is no general federal mandate requiring providers to collect SOGI data. Certified EHRs have been expected to support SOGI fields through USCDI standards, but federal regulators announced enforcement discretion in 2025 on SOGI-related certification elements^1^, further weakening de facto uptake (while some programs, e.g., HRSA-funded community health centers, retain specific SOGI reporting requirements). Concurrently, recent federal actions have reduced the availability or timeliness of equity-relevant data (including SOGI and race/ethnicity variables) across multiple datasets and websites, complicating disparity monitoring and accountability efforts [22]. These dynamics reinforce our choice to interpret WPHS findings as person-level signals and underscore the need for system-level data supports in operational deployments.

## Conclusion

We have demonstrated the effectiveness of an EHR-embedded tool in identifying non-medical needs for the LGB+ community. This tool highlights areas requiring targeted interventions with the goal of improving outcomes. Moreover, the WPHS tool is strategically crafted to promote collaboration among clinicians and other care team members, fostering a streamlined process for creating referrals when interventions are deemed essential. The extent to which these referrals, directed towards resources identified by our tool, contribute to enhanced health outcomes and improved overall well-being for patients within the LGB+ community remains a subject for further investigation. As we advance, future studies will play a pivotal role in gauging the impact and efficacy of these interventions. A rigorous examination of the outcomes from referrals generated by our tool will provide valuable insights into the potential. We have demonstrated the effectiveness of an EHR-embedded tool in identifying non-medical needs for the LGB+ community, underscoring the Whole Person Health (WPH) model’s relevance for addressing these needs comprehensively. This tool highlights areas requiring targeted interventions to improve outcomes and reflects the holistic perspective of WPH by considering mental, emotional, and social dimensions alongside physical health. The WPHS tool is strategically crafted to promote collaboration among clinicians and other care team members, fostering a streamlined process for creating referrals when interventions are deemed essential. The extent to which these referrals, directed towards resources identified by our tool, contribute to enhanced health outcomes and improved overall well-being for patients within the LGB+ community remains a subject for further investigation. Future studies will play a pivotal role in gauging the impact and efficacy of these interventions, providing valuable insights into the potential improvements in health outcomes and patient well-being. This avenue of research is essential for substantiating the real-world implications of the WPH model, as it offers a more comprehensive framework than traditional SDOH approaches for understanding and addressing disparities in the LGB+ community and guiding future developments in patient-centered care.

## Data Availability

The data used in this study are derived from electronic health records (EHRs) within a safety-net health system and include de-identified patient information. Due to the sensitive nature of patient data and institutional policies, access to the dataset is restricted. Researchers interested in accessing the data may submit a request to the corresponding author and must comply with the institution’s data-sharing agreements and ethical guidelines.

## Appendix

**Table A1:** The 28 dimensions associated with the six domains of the PERSON Score

Physical Health	High Blood Pressure Abnormal BMI Any Chronic Conditions Unable to Perform Functional Activity
Emotional Health	Felt depressed in the past month Felt anxious in the past month Lack of Social Support Lack of time to Pray/Meditate/Relax Lack of Meaning/Purpose
Resource Utilization	Any ER/Hospitalization Multiple Office-based Visits Any Prescription Meds
Socioeconomics	Inadequate Household Finances Unstable/Unsafe Housing Lack of Higher Education Unemployed or Disabled Inadequate Food Access Lack of Transportation
Ownership	Overall Status: Not Healthy Inadequate Knowledge of Own Health Negative Self-Efficacy Inadequate Self-management
Nutrition & Lifestyle	Unhealthy Eating Habits Lack of Physical Activity Inadequate Sleep Current or Past Smoker Adverse Substance Use

Appendix Table A2 (Part I): Difference in the probability of an adverse outcome among LGB+ and Others by Age Group and Gender. Notes: The reference group is heterosexual individuals of the same age or gender. Each column in each panel is a separate regression. Average marginal effects are presented. Standard errors are presented in parentheses, and 95% confidence intervals are presented in brackets. *** indicates *p* < 0.001, ** *p* < 0.01, * *p* < 0.05, + *p* < 0.1. Example Interpretation: Young (aged 18-25 years) LGB+ patients were 7.2 pp more likely to report an Abnormal BMI compared to their heterosexual counterparts.

**Table:** Part I

	Physical Health				Emotional Health					Resource Utilization		
High Blood Pressure	Abnormal BMI	Any Chronic Conditions	Unable to Perform Functional Activity	Felt depressed in the past month	Felt anxious in the past month	Lack of Social Support	Lack of time to Pray/Meditate/Relax	Lack of Clarity in Meaning/Purpose	Any ER / Hospitalization	Multiple Office-based Visits	Any Prescription Meds	
Panel 1: By Age Group												
18-25 LGB+	0.011	0.072**	0.088**	0.04	0.241***	0.229***	0.145***	0.076*	0.264***	0.015	0.043	0.135***
	(0.029)	(0.023)	(0.027)	(0.023)	(0.028)	(0.025)	(0.025)	(0.029)	(0.029)	(0.029)	(0.027)	(0.029)
	[-0.046,0.069]	[0.026,0.118]	[0.034,0.142]	[-0.004,0.084]	[0.187,0.296]	[0.180,0.278]	[0.096,0.193]	[0.019,0.134]	[0.207,0.321]	[-0.042,0.071]	[-0.011,0.097]	[0.078,0.192]
18-25 Other	0.04	0.035	0.193**	0.061	0.136*	0.078	0.042	0	0.233***	-0.023	0.035	0.316***
	(0.056)	(0.048)	(0.056)	(0.046)	(0.056)	(0.055)	(0.053)	(0.056)	(0.057)	(0.054)	(0.053)	(0.054)
	[-0.070,0.150]	[-0.058,0.129]	[0.083,0.304]	[-0.029,0.150]	[0.025,0.246]	[-0.030,0.187]	[-0.063,0.146]	[-0.110,0.111]	[0.122,0.344]	[-0.129,0.084]	[-0.069,0.138]	[0.209,0.422]
26-45 LGB+	-0.026	-0.005	0.068**	0.007	0.170***	0.166***	0.041*	0.013	0.157***	-0.001	0.074***	0.125***
	(0.020)	(0.015)	(0.020)	(0.017)	(0.020)	(0.019)	(0.019)	(0.020)	(0.019)	(0.020)	(0.020)	(0.019)
	[-0.065,0.014]	[-0.034,0.024]	[0.029,0.107]	[-0.027,0.040]	[0.132,0.209]	[0.130,0.203]	[0.004,0.078]	[-0.026,0.053]	[0.119,0.195]	[-0.040,0.037]	[0.035,0.114]	[0.087,0.163]
26-45 Other	0.072	0.021	0.128**	0.066	0.120**	0.071	0.04	-0.022	0.197***	0.058	0.064	0.156***
	(0.038)	(0.027)	(0.039)	(0.036)	(0.039)	(0.038)	(0.037)	(0.039)	(0.038)	(0.039)	(0.039)	(0.037)
	[-0.002,0.146]	[-0.031,0.074]	[0.051,0.205]	[-0.005,0.136]	[0.043,0.196]	[-0.005,0.146]	[-0.032,0.113]	[-0.098,0.054]	[0.121,0.272]	[-0.019,0.134]	[-0.012,0.140]	[0.083,0.228]
46+ LGB+	0.002	0.001	0.058*	-0.002	0.072*	0.101**	0.081**	0.035	0.160***	0.088**	0.058	0.057**
	(0.030)	(0.022)	(0.028)	(0.031)	(0.031)	(0.031)	(0.028)	(0.029)	(0.030)	(0.031)	(0.031)	(0.020)
	[-0.056,0.060]	[-0.042,0.044]	[0.002,0.113]	[-0.064,0.059]	[0.010,0.133]	[0.041,0.162]	[0.026,0.135]	[-0.023,0.092]	[0.101,0.219]	[0.026,0.149]	[-0.003,0.118]	[0.019,0.096]
46+ Other	-0.038	-0.083*	-0.018	-0.001	0.074	0.075	0.039	-0.004	0.108**	0.019	-0.011	0.059*
	(0.041)	(0.036)	(0.041)	(0.042)	(0.042)	(0.042)	(0.039)	(0.038)	(0.039)	(0.042)	(0.042)	(0.026)
	[-0.118,0.043]	[-0.153,-0.013]	[-0.098,0.062]	[-0.084,0.081]	[-0.009,0.157]	[-0.007,0.157]	[-0.038,0.116]	[-0.079,0.071]	[0.032,0.185]	[-0.063,0.100]	[-0.094,0.072]	[0.008,0.111]
N (Observations)	35,992	35,992	35,992	35,992	35,992	35,992	35,992	35,992	35,992	35,992	35,992	35,992
Panel II: By Gender												
Female LGB+	-0.031	0.021	-0.001	-0.02	0.180***	0.198***	0.065***	0.115***	0.237***	0.056**	0.015	0
	(0.019)	(0.013)	(0.019)	(0.018)	(0.019)	(0.017)	(0.018)	(0.019)	(0.019)	(0.019)	(0.019)	(0.019)
	[-0.069,0.007]	[-0.006,0.047]	[-0.039,0.037]	[-0.055,0.015]	[0.143,0.216]	[0.165,0.232]	[0.031,0.100]	[0.077,0.153]	[0.200,0.275]	[0.018,0.093]	[-0.023,0.053]	[-0.036,0.037]
Female Other	0.016	-0.032	0.090**	0.01	0.132***	0.099**	0.022	0.001	0.210***	0.021	0.009	0.095**
	(0.033)	(0.027)	(0.034)	(0.032)	(0.034)	(0.033)	(0.032)	(0.033)	(0.033)	(0.033)	(0.034)	(0.030)
	[-0.049,0.082]	[-0.084,0.021]	[0.024,0.156]	[-0.053,0.073]	[0.066,0.197]	[0.035,0.163]	[-0.041,0.086]	[-0.062,0.065]	[0.145,0.275]	[-0.044,0.086]	[-0.057,0.075]	[0.037,0.154]
Male LGB+	-0.082***	-0.035	-0.101***	-0.131***	0.160***	0.174***	0.072***	0.023	0.127***	-0.028	-0.021	-0.032
	(0.022)	(0.018)	(0.022)	(0.019)	(0.022)	(0.021)	(0.020)	(0.022)	(0.021)	(0.022)	(0.022)	(0.021)
	[-0.124,-0.039]	[-0.069,0.000]	[-0.144,-0.057]	[-0.167,-0.094]	[0.117,0.204]	[0.132,0.216]	[0.033,0.111]	[-0.019,0.066]	[0.085,0.169]	[-0.070,0.014]	[-0.064,0.022]	[-0.074,0.009]
Male Other	-0.023	-0.019	-0.059	-0.02	0.083*	0.07	0.052	0.017	0.123**	0.028	-0.015	0.072*
	(0.037)	(0.030)	(0.039)	(0.037)	(0.039)	(0.039)	(0.036)	(0.038)	(0.038)	(0.039)	(0.039)	(0.034)
	[-0.095,0.050]	[-0.078,0.040]	[-0.136,0.017]	[-0.092,0.053]	[0.006,0.160]	[-0.007,0.146]	[-0.017,0.122]	[-0.058,0.091]	[0.049,0.196]	[-0.048,0.104]	[-0.090,0.061]	[0.007,0.138]
N (Observations)	35,992	35,992	35,992	35,992	35,992	35,992	35,992	35,992	35,992	35,992	35,992	35,992

Appendix Table A2 (Part II):

**Table:** Part II

	Socioeconomics						Ownership				Nutrition & Lifestyle				
Inadequate Household Finances	Unstable/Unsafe Housing	Lack of Higher Education	Unemployed or Disabled	Inadequate Food Access	Lack of Transportation	Overall Status: Not Healthy	Inadequate Knowledge of Own Health	Negative Self-Efficacy	Inadequate Self-management	Unhealthy Eating Habits	Lack of Physical Activity	Inadequate Sleep	Current or Past Smoker	Adverse Substance Use	
Panel 1: By Age Group															
18-25 LGB+	0.215***	0.029**	-0.012	0.057*	0.160***	0.116***	0.124***	0.085**	0.125***	0.147***	0.190***	0.090**	0.172***	0.063**	0.044*
	(0.029)	(0.011)	(0.028)	(0.027)	(0.028)	(0.026)	(0.028)	(0.027)	(0.027)	(0.029)	(0.029)	(0.028)	(0.024)	(0.021)	(0.019)
	[0.159,0.271]	[0.007,0.050]	[-0.067,0.044]	[0.003,0.110]	[0.106,0.214]	[0.065,0.167]	[0.069,0.179]	[0.032,0.139]	[0.073,0.177]	[0.091,0.204]	[0.133,0.246]	[0.034,0.146]	[0.125,0.219]	[0.022,0.105]	[0.008,0.081]
18-25 Other	0.091	-0.01***	0	0.156**	0.057	0.117*	0.062	-0.021	0.079	0.02	0.078	0.189**	0.069	-0.02	-0.01
	(0.057)	(0.002)	(0.054)	(0.056)	(0.049)	(0.051)	(0.052)	(0.047)	(0.049)	(0.057)	(0.057)	(0.057)	(0.052)	(0.030)	(0.028)
	[-0.020,0.203]	[-0.013,-0.006]	[-0.106,0.107]	[0.046,0.266]	[-0.038,0.152]	[0.018,0.217]	[-0.041,0.164]	[-0.113,0.071]	[-0.018,0.176]	[-0.091,0.131]	[-0.033,0.190]	[0.078,0.300]	[-0.034,0.171]	[-0.079,0.039]	[-0.065,0.044]
26-45 LGB+	0.133***	0.028**	-0.115***	0.101***	0.108***	0.105***	0.041*	0.007	0.044**	0.086***	0.084***	0.035	0.097**	0.117***	0.067***
	(0.019)	(0.009)	(0.020)	(0.019)	(0.019)	(0.018)	(0.020)	(0.018)	(0.017)	(0.020)	(0.020)	(0.019)	(0.018)	(0.019)	(0.016)
	[0.096,0.170]	[0.010, 0.046]	[-0.154,-0.075]	[0.064,0.139]	[0.070,0.145]	[0.069,0.140]	[0.002,0.079]	[-0.029,0.043]	[0.011,0.077]	[0.046,0.125]	[0.045,0.124]	[-0.003,0.072]	[0.063,0.132]	[0.079,0.155]	[0.035,0.098]
26-45 Other	0.038	0.051*	-0.008	0.158***	0.096**	0.062	0.035	0.019	0.068*	0.05	0.048	0.098*	0.049	0.074*	0.070*
	(0.039)	(0.021)	(0.038)	(0.039)	(0.037)	(0.033)	(0.038)	(0.036)	(0.034)	(0.039)	(0.039)	(0.038)	(0.036)	(0.037)	(0.031)
	[-0.038,0.114]	[0.010, 0.093]	[-0.083,0.067]	[0.082,0.234]	[0.024,0.168]	[-0.003,0.127]	[-0.040,0.110]	[-0.052,0.090]	[0.002,0.134]	[-0.027,0.126]	[-0.029,0.125]	[0.022,0.173]	[-0.021,0.120]	[0.001,0.146]	[0.008,0.132]
46+ LGB+	0.05	0.036*	-0.243***	0.095**	0.068*	0.034	0.012	-0.064*	0.01	0.082**	0.056	0.021	0.078**	0.255***	0.057*
	(0.029)	(0.016)	(0.031)	(0.031)	(0.030)	(0.027)	(0.031)	(0.028)	(0.026)	(0.030)	(0.031)	(0.030)	(0.027)	(0.031)	(0.024)
	[-0.007,0.108]	[0.004, 0.068]	[-0.304,-0.181]	[0.033,0.156]	[0.009,0.126]	[-0.019,0.086]	[-0.050,0.074]	[-0.120,-0.008]	[-0.040,0.060]	[0.023,0.141]	[-0.005,0.118]	[-0.038,0.080]	[0.024,0.132]	[0.194,0.316]	[0.009,0.104]
46+ Other	-0.012	0.005	-0.158***	0.026	0.067	0.006	0	-0.017	-0.001	0.023	-0.077*	0.004	-0.018	0.098*	0.048
	(0.041)	(0.017)	(0.042)	(0.042)	(0.040)	(0.034)	(0.042)	(0.040)	(0.034)	(0.039)	(0.039)	(0.040)	(0.040)	(0.042)	(0.032)
	[-0.093,0.069]	[-0.029, 0.038]	[-0.240,-0.077]	[-0.056,0.107]	[-0.012,0.146]	[-0.061,0.074]	[-0.084,0.083]	[-0.095,0.061]	[-0.067,0.066]	[-0.052,0.099]	[-0.153,-0.001]	[-0.075,0.083]	[-0.097,0.061]	[0.015,0.180]	[-0.014,0.111]
N (Observations)	35,992	35,992	35,992	35,992	35,992	35,992	35,992	35,992	35,992	35,992	35,992	35,992	35,992	35,992	35,992
Panel II: By Gender															
Female LGB+	0.123***	0.032***	-0.112***	0.093***	0.124***	0.111***	0.064**	0.023	0.093***	0.208**	0.184***	0.053**	0.134***	0.129***	0.075***
	(0.018)	(0.009)	(0.019)	(0.019)	(0.018)	(0.017)	(0.019)	(0.018)	(0.017)	(0.019)	(0.019)	(0.019)	(0.015)	(0.018)	(0.014)
	[0.088,0.159]	[0.015,0.049]	[-0.150,-0.074]	[0.057,0.130]	[0.088,0.160]	[0.077,0.145]	[0.026,0.102]	[-0.013,0.059]	[0.059,0.127]	[0.170,0.246]	[0.147,0.222]	[0.017,0.090]	[0.104,0.164]	[0.093,0.164]	[0.048,0.103]
Female Other	0.036	0.008	-0.090**	0.078*	0.063*	0.052	0.032	-0.003	0.029	0.096***	0.042	0.049	0.056	0.077*	0.070**
	(0.034)	(0.012)	(0.034)	(0.032)	(0.031)	(0.028)	(0.034)	(0.031)	(0.028)	(0.034)	(0.034)	(0.033)	(0.030)	(0.031)	(0.024)
	[-0.030,0.102]	[-0.015,0.032]	[-0.156,-0.024]	[0.015,0.142]	[0.002,0.123]	[-0.003,0.107]	[-0.035,0.098]	[-0.064,0.059]	[-0.027,0.084]	[0.030,0.162]	[-0.024,0.108]	[-0.016,0.113]	[-0.003,0.115]	[0.017,0.137]	[0.023,0.118]
Male LGB+	0.024	0.009	-0.221***	-0.026	0.039	0.034	-0.103***	-0.063**	-0.004	0.066**	0.024	0.005	0.075***	-0.017	-0.007
	(0.021)	(0.010)	(0.022)	(0.022)	(0.021)	(0.019)	(0.021)	(0.020)	(0.017)	(0.022)	(0.022)	(0.021)	(0.020)	(0.022)	(0.018)
	[-0.017,0.066]	[-0.011,0.029]	[-0.264,-0.177]	[-0.068,0.017]	[-0.002,0.079]	[-0.004,0.072]	[-0.144,-0.062]	[-0.102,-0.025]	[-0.037,0.030]	[0.023,0.109]	[-0.019,0.067]	[-0.035,0.046]	[0.035,0.115]	[-0.059,0.026]	[-0.042,0.028]
Male Other	-0.055	0.027	-0.076*	0.084*	0.059	0.032	-0.059	-0.036	0.060*	0.006	-0.024	0.113**	-0.009	-0.062	-0.026
	(0.039)	(0.020)	(0.037)	(0.039)	(0.037)	(0.034)	(0.038)	(0.035)	(0.034)	(0.037)	(0.038)	(0.039)	(0.038)	(0.037)	(0.030)
	[-0.131,0.021]	[-0.013,0.067]	[-0.149,-0.003]	[0.007,0.161]	[-0.013,0.132]	[-0.034,0.099]	[-0.134,0.015]	[-0.106,0.033]	[-0.005,0.126]	[-0.067,0.079]	[-0.098,0.049]	[0.037,0.189]	[-0.084,0.066]	[-0.135,0.012]	[-0.085,0.033]
N (Observations)	35,992	35,992	35,992	35,992	35,992	35,992	35,992	35,992	35,992	35,992	35,992	35,992	35,992	35,992	35,992

## Footnotes

1 https://www.healthit.gov/topic/uscdi-v3-data-elements-enforcement-discretion-notice
